# The mediating role of emotion regulation in the relationship between parental psychological control and psychological distress among students with smartphone addiction

**DOI:** 10.3389/fpsyt.2026.1680811

**Published:** 2026-02-10

**Authors:** Liu Xu, Zarinah Arshat, Nellie Ismail, Shi Lulu

**Affiliations:** 1 School of Educational Sciences, Xinxiang University, Henan, China; 2 Department of Human Development and Family Studies, Faculty of Human Ecology Universiti Putra Malaysia, Selangor Darul Ehsan, Malaysia; 3 Family, Adolescent and Child Research Centre of Excellence (FACE), Faculty of Human Ecology, Universiti Putra Malaysia, Selangor Darul Ehsan, Malaysia; 4 Department of Educational Psychology, School of Education, Weifang University of Science and Technology, Weifang, Shandong, China

**Keywords:** emotion regulation, parental psychological control, PLS-SEM, psychological distress, smartphone addiction

## Abstract

**Objectives:**

The purpose of this research was to investigate how emotion regulation, particularly reappraisal and inhibition, mediate the connection between psychological control from parents and psychological distress experienced by university students who are addicted to smartphones.

**Methods:**

A total of 1,276 university students from Henan Province, China, participated in this study. The proposed relationships and mediation pathways were examined through data analysis employing Partial Least Squares Structural Equation Modeling (PLS-SEM).

**Results:**

Both mother psychological control (β = 0.162, p < 0.05) and father psychological control (β = 0.319, p < 0.05) were positively associated with psychological distress. Reappraisal was negatively related to psychological distress (β = -0.074, p < 0.05), whereas inhibition showed a positive correlation (β = 0.299, p < 0.05). Father psychological control was positively associated with both reappraisal (β = 0.130, p < 0.05) and inhibition (β = 0.233, p < 0.05). Mother psychological control was positively linked to inhibition (β = 0.106, p < 0.05) but not to reappraisal (β = -0.036, p > 0.05). Inhibition significantly mediated the effects of father (β = 0.070, p < 0.05) and mother psychological control (β = 0.032, p < 0.05) on psychological distress, while reappraisal did not exhibit a mediation effect.

**Conclusion:**

These findings show that inhibition mediates the psychological effects of parental psychological control on university students with smartphone addiction. These connections were not mediated by reappraisal. Interventions to reduce parental psychological control-related psychological distress should target inhibitory mechanisms, according to the study.

## 1 Introduction

Smartphone addiction has increasingly been recognized as a global public health concern, with significant implications for psychological well-being across diverse populations, particularly in China where prevalence rates continue to escalate (1). Empirical research consistently indicates that individuals suffering from smartphone addiction are at heightened risk of developing mental health disorders, especially psychological distress (2). Psychological distress encompasses a range of cognitive, emotional, and behavioral symptoms, including persistent anxiety, depression, tension, and feelings of hopelessness (3). According to a joint report by the World Health Organization (3), approximately one in five adolescents worldwide experiences psychological distress, underscoring its importance as a critical public health issue.

University students are particularly vulnerable to psychological distress stemming from smartphone overuse due to the interplay of biological predispositions, psychological vulnerabilities, and socio-ecological stressors (4). One of these characteristics that has recently come to light as a strong indicator of psychological maladjustment in young adults is parental psychological control (5). Psychological control refers to a pattern of parenting characterized by intrusive practices that undermine adolescents’ emotional and psychological autonomy (6). This form of control typically involves covert regulatory strategies, including guilt induction, conditional expressions of affection, and excessive parental oversight, which can interfere with the development of a coherent identity and emotional self-governance. As many university students remain emotionally and financially reliant on their parents (7), exposure to psychologically controlling parenting may be especially detrimental during this developmental period. Empirical research has associated parental psychological control with diminished autonomy (8), elevated psychological distress (9), and a greater susceptibility to smartphone addiction (10).

Growing evidence suggests that the association between smartphone addiction and psychological distress is bidirectional, such that excessive smartphone use increases vulnerability to depression and distress, while impaired mental health may also predispose individuals to develop smartphone addiction, including depressive symptoms predicting later addictive use (11) and maladaptive parenting influencing well-being indirectly through self-esteem in young adults (12). However, most prior studies have primarily emphasized psychological problems as antecedents of smartphone addiction. In contrast, the present study adopts a complementary perspective by examining how parental psychological control and emotion regulation processes contribute to psychological distress within an already smartphone addicted population, thereby offering a more balanced understanding of the interplay between family dynamics, emotional regulation, and mental health among risk individuals.

In parallel, an expanding body of research has documented alarming prevalence rates of technology-related behavioral addictions among university students. Eichenberg et al. (13) reported that 22.7% of university students met the criteria for social media addiction, which was significantly associated with higher levels of anxiety, somatic symptoms, and psychological distress. Similarly, Zhang et al. (14) discovered that 49.5% of Chinese medical students were classed as addicted to smartphones, and that these students showed much more psychological distress than their non-addicted classmates. Notably, 79.4% of smartphone-addicted students reported severe psychological distress, in stark contrast to the 20.6% distress rate among non-addicted individuals.

These findings highlight the increasing mental health burden linked to excessive engagement with smartphones and social media, underscoring the need to further investigate the underlying psychological mechanisms involved. A significant external stressor is parental psychological control, but intrapersonal regulatory processes appear to play a pivotal mediation role in explaining individual variances in emotional coping (15). The term “emotion regulation” describes the methods people use to control their feelings and the way in which they react to stressful situations. Adaptive strategies, such as cognitive reappraisal, allow individuals to reinterpret emotional events, thereby mitigating distress. On the flip side, emotional loads and psychological distress are both exacerbated by maladaptive methods like emotion repression (16).

There is mounting evidence that emotions regulate and mediate the connection between environmental stresses and psychological consequences. Mohammadkhani et al. (17) demonstrated that emotion regulation mediates the influence of negative emotions on anxiety and depression. Similarly, O’Rourke and Egan (18) found that emotion regulation processes mediate the link between insecure attachment and psychological distress. According to Ma, Wang, and Gao (19), university students in China showed a correlation between parental psychological control and psychological resilience through inhibition rather than reappraisal. According to these results, methods for controlling one’s emotions can mitigate or worsen the effect of psychological control from parents on their children’s mental health. In particular, expression inhibition has been consistently linked to heightened distress in adverse social environments (20).

Ecological Systems Theory posits that parental psychological control within the microsystem hinders emotion regulation, which mediates its impact on psychological distress (21). The individual’s maladaptive regulation strategies reflect the interplay between family dynamics and broader socio-environmental influences on mental health.

Despite extensive research on parental psychological control, emotion regulation, and psychological distress, important theoretical and empirical gaps remain. Although parental psychological control has been widely examined during childhood and adolescence, its role during emerging adulthood has received limited attention, despite university students often remaining emotionally and financially dependent on their parents, particularly in collectivistic cultural contexts such as China. Moreover, prior research has frequently conceptualized emotion regulation as a global construct, thereby overlooking meaningful distinctions between specific strategies. To overcome this gap, the current investigation differentiates between cognitive reappraisal and expressive suppression and evaluates their respective mediating functions in the relationship between parental psychological control and psychological distress. In addition, most existing studies have focused on general university populations, with relatively little attention devoted to students with smartphone addiction, a risk group characterized by heightened emotional vulnerability and maladaptive coping tendencies. Finally, although parental psychological control is increasingly recognized as a multidimensional construct, few studies have systematically differentiated between paternal and maternal psychological control, particularly in the context of digital behavioral addictions. Addressing these gaps, the present study aims to clarify how parental psychological control influences psychological distress among smartphone addicted university students through specific emotion regulation, while accounting for potential differences between paternal and maternal influences.

Previous studies suggest that the psychological impact of parenting is context-dependent. For example, Zhang, He, and Xu (22) demonstrated that parenting effects differ by immigration background, providing a conceptual rationale for considering students with smartphone addiction as a distinct group characterized by heightened contextual vulnerability.

**Figure 1:**
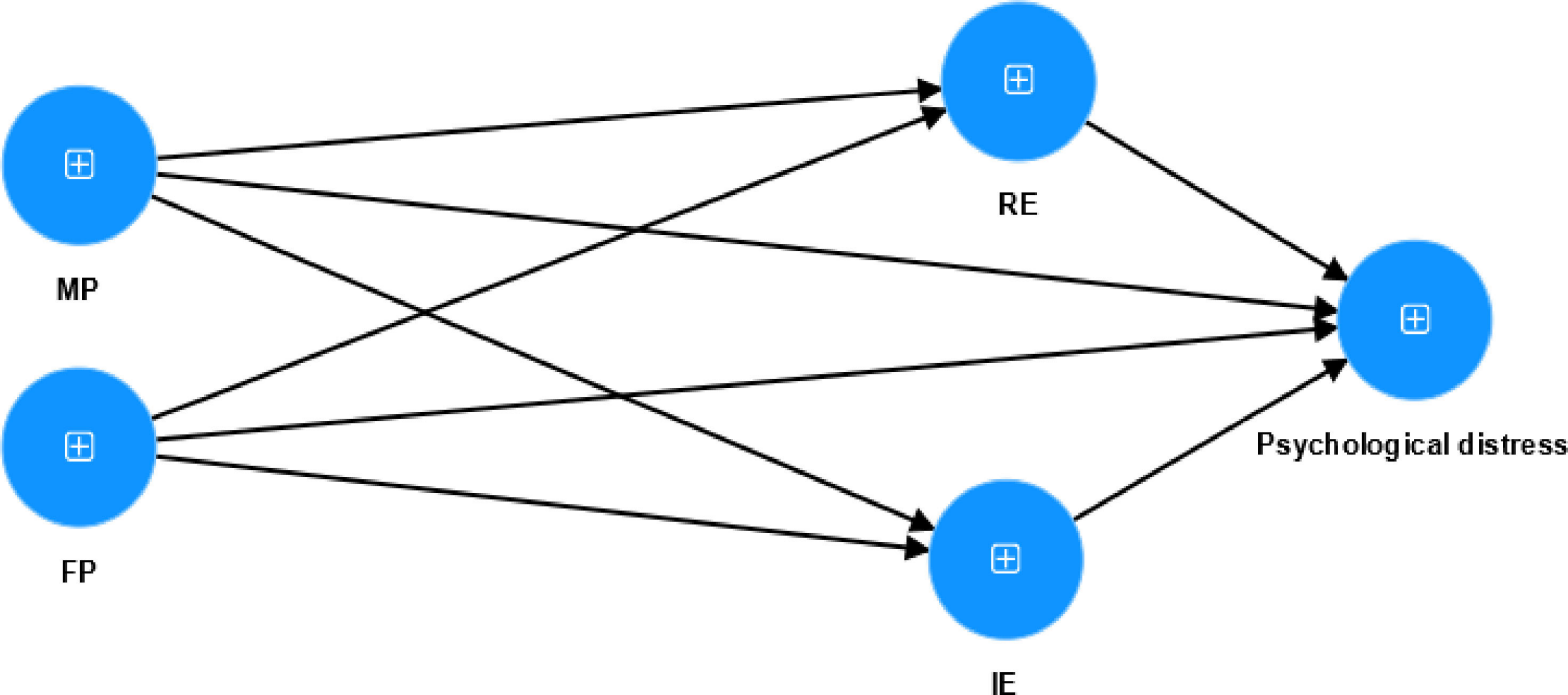
Study framework. MP, Mother Psychological Control; FP, Father Psychological Control; RE, Reappraisal; IE, Inhibition; PD, Psychological Distress. Abbreviations apply throughout.

The present study aims to clarify the mechanisms linking parental psychological control to psychological distress among university students with smartphone addiction by examining the mediating roles of emotion regulation. Specifically, this research investigates how parental psychological control influences students’ emotional coping processes and whether reappraisal and inhibition function as distinct pathways through which such parental psychological control contributes to psychological distress. In addition, the study differentiates between paternal and maternal psychological control to better capture the complexity of family influences on emotional maladjustment in this risk population. Grounded in ecological systems, the proposed conceptual framework outlining these hypothesized relationships is presented in **Figure 1**.

## 2 Method

### 2.1 Research design and participants

A cross-sectional quantitative survey was conducted to investigate the relationships between parental psychological control and psychological distress among university students exhibiting smartphone addiction, with emotion regulation tested as potential mediators. Maternal and paternal psychological control were specified as predictor variables, emotion regulation strategies were modeled as mediators, and psychological distress served as the outcome variable. Data were collected in May 2024 from public universities located in Henan Province, China.

Participants were recruited using purposive sampling based on smartphone addiction criteria. A total of 2162 university students initially participated in the survey. Among them, 1276 students met the criteria for smartphone addiction and were retained for subsequent analyses, yielding a detection rate of 59%. Classification of smartphone addiction was based on threshold values derived from the Smartphone Addiction Scale–Short Version, applying sex-specific cutoffs of ≥31 for men and ≥33 for women, as recommended by Kwon (23). The effective response rate for the retained sample was 100 percent. Missing data accounted for less than 5 percent of the dataset and were handled using mean substitution.

The final sample consisted of students aged between 18 and 24 years. Both male and female students were included, with a relatively balanced gender distribution. Participants represented all undergraduate year levels, including freshmen, sophomores, juniors, and seniors. Academic backgrounds were diverse and included students from humanities and social science disciplines as well as science and engineering related fields. An *a priori* power analysis conducted using G Power indicated that a minimum sample size of 107 participants was required to detect medium effect sizes with adequate statistical power (24). The achieved sample size substantially exceeded this requirement. According to established guidelines suggesting that samples larger than 1000 are considered excellent for empirical research, the current sample demonstrated strong statistical adequacy (25).

### 2.2 Instruments

All measurement instruments were administered using validated Chinese versions of the original scales. Previous research has demonstrated satisfactory reliability and validity of these Chinese versions in similar populations.

#### 2.2.1 Measurement of smartphone addiction

To evaluate participants’ tendencies toward smartphone addiction, this study employed an instrument comprising 10 items that assess problematic smartphone use behaviors. Items were rated using a six-point agreement scale, ranging from 1 (strongly disagree) to 6 (strongly agree), with higher summed scores reflecting more pronounced addictive tendencies. The tool applied cut-off thresholds of 31 for male participants and 33 for female participants to distinguish individuals at risk of addiction, following criteria established by Kwon et al. (23). The Chinese version of this scale has demonstrated good psychometric properties in prior research (26). In the present study, the scale showed satisfactory internal consistency, as reflected by a Cronbach’s alpha of 0.896.

#### 2.2.2 Assessment of psychological distress

General psychological distress was measured by summing responses to 10 items that capture the frequency of depressive and anxiety-related symptoms experienced over the past month. Each item was scored on a 5-point Likert scale, with response options ranging from “none of the time” (1) to “all of the time” (5). Total scores ranged between 10 and 50, where higher values corresponded to more intense and frequent experiences of psychological distress. The instrument used for this assessment was the Kessler Psychological Distress Scale (K10), originally developed by Kessler (27). Prior research has established the sound psychometric properties of the Chinese version of this instrument (28). In the current sample, internal consistency was excellent, with a Cronbach’s alpha of 0.935.

#### 2.2.3 Evaluation of parental psychological control

Participants’ subjective perceptions of how their parents exert psychological control were captured using an instrument designed to assess intrusive parenting behaviors that compromise a child’s emotional autonomy. This instrument consists of eight items rated on a five-point scale ranging from 1 (never true) to 5 (always true). The items capture psychologically controlling parenting behaviors, including guilt induction, emotional invalidation, and affection withdrawal. Originally developed by Barber (6), the measure permits independent assessment of paternal and maternal psychological control. Prior research has confirmed the reliability and validity of the Chinese adaptation (29). In the present sample, the scale demonstrated excellent internal consistency, with a Cronbach’s alpha of 0.930.

#### 2.2.4 Measurement of emotion regulation

Emotion regulation was assessed through a questionnaire that distinguishes between two key strategies: reappraisal and inhibition. The scale consists of 10 items, including six assessing cognitive reappraisal and four assessing expressive inhibition. Participants responded to all items using a seven-point Likert format, ranging from 1 (strongly disagree) to 7 (strongly agree). Developed by Gross and John (30), this questionnaire assesses individual differences in emotion regulation strategies across situational contexts. The psychometric adequacy of the Chinese version has been supported by previous studies (31). In the current study, internal consistency was acceptable, with Cronbach’s alpha coefficients of 0.878 for cognitive reappraisal and 0.842 for expressive inhibition.

### 2.3 Participants and sampling procedure

Participants were selected using a purposive sampling approach based on smartphone addiction status. A total of 2,162 university students were initially recruited. Smartphone addiction was determined using the Smartphone Addiction Scale–Short Version, applying cutoff scores of 31 for males and 33 for females as recommended by Kwon et al. (23). Based on these criteria, 1,276 students qualified for inclusion and were retained for subsequent analyses, corresponding to an estimated prevalence of approximately 59% within the original sample. The response rate for valid questionnaires was 100 percent, and missing data accounted for less than 5 percent of all responses. Given the low proportion of missing values, mean substitution was applied to handle missing data.

The final sample consisted of 1,276 university students aged 18 years and older, with a mean age of 19.94 years and a standard deviation of 1.34. Most participants were between 20 and 21 years old. The gender distribution was balanced, with 637 males representing 49.9 percent of the sample and 639 females representing 50.1 percent. Students from all undergraduate years were included, with sophomores comprising 35.7 percent of the sample, followed by juniors at 25.9 percent, freshmen at 25.3 percent, and seniors at 13.1 percent. Regarding academic background, 49.37 percent of participants were enrolled in arts related disciplines and 50.63 percent in science related disciplines.

### 2.4 Data analysis

Data analyses were conducted using SPSS and SmartPLS software. SPSS was employed to compute descriptive statistics and conduct preliminary analyses. Partial Least Squares Structural Equation Modeling was applied using SmartPLS version 4 to test the proposed measurement and structural models, following the analytical procedures outlined by Ringle et al. (32). PLS SEM was selected due to its robustness in estimating complex models with multiple latent constructs, its tolerance for non-normal data distributions, and its suitability for psychological research involving diverse measurement scales (33, 34). All latent variables in the present study were specified as reflective constructs, consistent with the theoretical assumptions underlying the measured psychological processes.

Mediation effects were examined using a bootstrapping procedure with 5,000 resamples. Indirect effects were considered statistically significant when the 95 percent confidence intervals did not include zero, in accordance with the recommendations of Preacher and Hayes (35). To address potential concerns regarding common method variance, several procedural remedies were implemented during data collection, and Harman single factor test was conducted as a diagnostic assessment. Consistent with the criteria proposed by Podsakoff et al. (36, 37), the results indicated that no single factor accounted for the majority of variance, suggesting that common method bias was unlikely to substantially influence the observed relationships.

## 3 Results

### 3.1 Preliminary analyses

#### 3.1.1 Common method variance

To evaluate the extent of common method variance (CMV) within the dataset, Harman’s single-factor test was conducted (36). The analysis revealed that the variance explained by the first unrotated component was 37.30 percent, which is considerably lower than the generally accepted threshold of 50 percent (38). This outcome indicates that common method bias does not pose a significant threat to the validity of the study’s results. Therefore, concerns regarding systematic error from a single data source were minimal, affirming the robustness of the analytical findings and confirming that method bias did not heavily influence the observed relationships.

#### 3.1.2 Descriptive statistics and correlations

Descriptive statistics for the primary study variables are presented in **Table 1**. The mean score for paternal psychological control was 2.28 (SD = 1.00), while maternal psychological control showed a slightly higher mean of 2.36 (SD = 0.89). The average level of expressive inhibition was 4.10 (SD = 1.14), and cognitive reappraisal demonstrated a relatively higher mean of 4.96 (SD = 0.87). Psychological distress had a mean score of 2.36 (SD = 0.89). The observed minimum and maximum values for all variables were consistent with the theoretical ranges of their respective measurement scales, indicating appropriate scale utilization.

**Table 1:** Descriptive statistics among key variables.

Variables	N	M	SD	Min	Max
FP	1276	2.28	1.00	1	5
MP	1276	2.36	0.89	1	4.63
IE RE	1276	4.10	1.14	1	7
	1276	4.96	0.87	3	7
PD	1276	2.36	0.89	1	4.6

Bivariate correlations among the study variables are reported in **Table 2**. All correlations were positive and statistically significant. Paternal and maternal psychological control were strongly associated with each other (r = 0.796, p < 0.01). Paternal psychological control was positively correlated with expressive inhibition (r = 0.314, p < 0.01), cognitive reappraisal (r = 0.096, p < 0.05), and psychological distress (r = 0.533, p < 0.01). Similarly, maternal psychological control showed significant positive associations with expressive inhibition (r = 0.284, p < 0.01), cognitive reappraisal (r = 0.063, p < 0.05), and psychological distress (r = 0.490, p < 0.01). Expressive inhibition was moderately and positively correlated with psychological distress (r = 0.422, p < 0.01), whereas cognitive reappraisal demonstrated a relatively small yet statistically significant positive association with psychological distress (r = 0.060, p < 0.05). Skewness and kurtosis values (-0.459 - 0.627) for all variables were within acceptable ranges, indicating no substantial deviations from normality.

**Table 2:** Correlations between variables.

Variables	*M ± SD*	*Skewness*	*Kurtosis*	1	2	3	4
1.FP	2.28 ± 1.00	0.627	-0.221				
2.MP	2.36 ± 0.89	0.450	-0.459	0.796^**^			
3.IE	4.10 ± 1.14	0.011	0.297	0.314^**^	0.284^**^		
4.RE	4.96 ± 0.87	0.529	-0.205	0.096^**^	0.063^*^	0.334^**^	
5.PD	2.36 ± 0.89	0.544	-0.451	0.533^**^	0.490^**^	0.422^**^	0.060^*^

*p <0.05 (two-tailed). **p <0.01 (two-tailed).

Taken together, these descriptive and correlational findings provide preliminary support for the hypothesized relationships among parental psychological control, emotion regulation strategies, and psychological distress. These results justify the subsequent evaluation of the measurement model prior to testing the proposed structural relationships.

### 3.2 Measurement model results

The measurement model was assessed using Partial Least Squares–Structural Equation Modeling (PLS-SEM) implemented in SmartPLS. This variance-based approach was selected due to its suitability for complex models, heterogeneous measurement scales, and potential deviations from multivariate normality (33, 34). All latent constructs were specified as reflective, and the measurement model comprised 36 observed indicators representing five latent variables.

Prior to examining the structural relationships, the measurement model was assessed to ensure adequate reliability and validity of all constructs. Internal consistency reliability was supported, as Cronbach’s alpha coefficients for all latent variables exceeded the recommended minimum threshold, indicating satisfactory internal consistency in accordance with established psychometric guidelines (34, 39). In addition, composite reliability values for all constructs surpassed the recommended criteria for PLS SEM, providing further evidence of construct reliability (33).

Convergent validity was assessed through an inspection of item loadings and average variance extracted (AVE) values. All items exhibited statistically significant loadings of adequate strength, indicating that the indicators reliably captured their intended latent constructs. In addition, AVE estimates for all constructs surpassed the threshold of 0.50, suggesting that a substantial proportion of variance in the indicators was accounted for by the underlying constructs, thereby providing evidence of acceptable convergent validity (40). The corresponding statistics are reported in **Table 3**.

**Table 3:** Loadings, CR and AVE values for low-order components.

Components	Loading	Composite Reliability (CR)	Average Variance Extracted (AVE)
Psychological Distress		0.956	0.685
PD1	0.745		
PD2	0.782		
PD3	0.836		
PD4	0.843		
PD5	0.814		
PD6	0.853		
PD7	0.839		
PD8	0.877		
PD9	0.854		
PD10	0.823		
Parental psychological control			
Mother Psychological Control		0.933	0.635
MP1	0.752		
MP2	0.747		
MP3	0.832		
MP4	0.846		
MP5	0.764		
MP6	0.808		
MP7	0.830		
MP8	0.787		
Father Psychological Control		0.950	0.703
FP1	0.809		
FP2	0.803		
FP3	0.879		
FP4	0.862		
FP5	0.820		
FP6	0.864		
FP7	0.841		
FP8	0.827		
Emotion regulation			
Reappraisal		0.901	0.604
RE1	0.800		
RE2	0.748		
RE3	0.711		
RE4	0.782		
RE5	0.811		
RE6	0.807		
Inhibition		0.864	0.615
IE1	0.799		
IE2	0.697		
IE3	0.827		
IE4	0.809		

Composite reliability values greater than 0.70 indicate satisfactory internal consistency reliability, and average variance extracted values exceeding 0.50 demonstrate adequate convergent validity. All indicator loadings were statistically significant and exceeded the recommended minimum value of 0.70, supporting the reliability and validity of the measurement model.

Discriminant validity was assessed using both the Fornell Larcker criterion and the heterotrait monotrait ratio of correlations. According to the Fornell Larcker criterion, the square root of the average variance extracted for each construct was greater than its correlations with all other constructs, indicating adequate discriminant validity (40). As shown in **Table 4**, all diagonal values exceeded the corresponding off diagonal correlations. In addition, heterotrait monotrait ratios were examined as a more stringent test of discriminant validity. All HTMT values were well below the conservative threshold of 0.90, further confirming satisfactory discriminant validity among the latent constructs (41), as reported in **Table 5**.

**Table 4:** Discriminant validity - Fornell–Larcker criterion.

Variables	1	2	3	4	5
(1) FP	0.838				
(2) MP	0.799	0.797			
(3) IE	0.318	0.292	0.784		
(4) RE	0.099	0.066	0.332	0.777	
(5) PD	0.536	0.499	0.423	0.067	0.827

Discriminant validity is established when √AVE > interconstruct correlations.

**Table 5:** HTMT criterion of higher order constructs of Model.

Variables	1	2	3	4
(1) FP				
(2) MP	0.854			
(3) IE	0.365	0.333		
(4) RE	0.108	0.073	0.400	
(5) PD	0.562	0.521	0.484	0.076

Discriminant validity established when HTMT <0.90.

For clarity, observed indicators were coded as PD1 through PD10 for psychological distress, MP1 through MP8 for maternal psychological control, FP1 through FP8 for paternal psychological control, RE1 through RE8 for cognitive reappraisal, and IE1 through IE4 for expressive inhibition.

### 3.3 Structural model results

**Figure 2:**
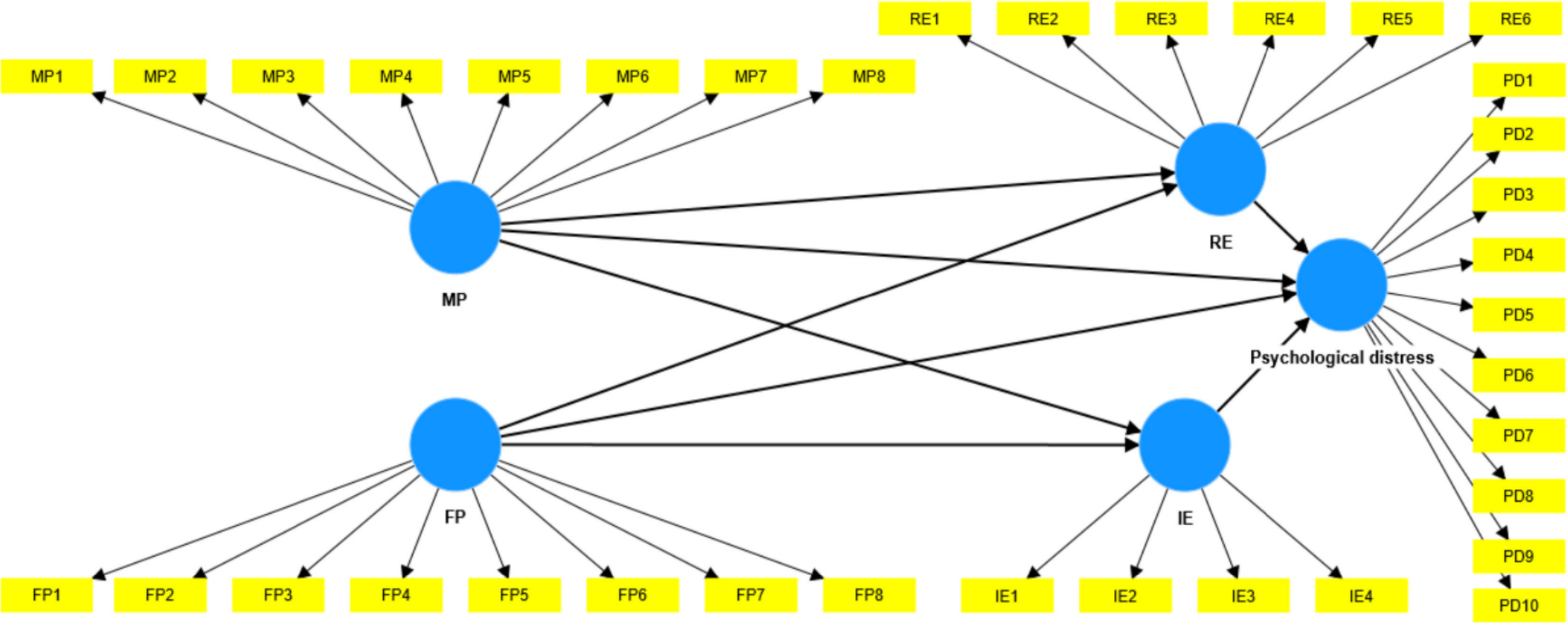
Post analyses model.

After establishing the adequacy of the measurement model, the structural relationships among the latent constructs were examined. The structural model was evaluated by inspecting explained variance, standardized path coefficients, t values, significance levels, variance inflation factors, and bootstrap confidence intervals. The results of the structural model analysis are summarized in **Table 6**, and the final model is illustrated in **Figure 2**. Collinearity diagnostics indicated no multicollinearity concerns, as all inner variance inflation factor values were below the recommended threshold of 3.0 and did not exceed the maximum observed value of 2.827 (33).

**Table 6:** Results of PLS-SEM analysis.

Paths	β	t-value	p-value	95% CI	VIF
Direct effects					
MP -> PD	0.162	3.943	**<0.001**	[0.079, 0.241]	2.782
FP -> PD	0.319	7.601	**<0.001**	[0.238, 0.402]	2.827
RE -> PD	-0.074	2.845	**0.004**	[-0.124, -0.023]	1.127
IE -> PD	0.299	10.826	**<0.001**	[0.243, 0.352]	1.246
MP -> RE	-0.038	0.635	0.526	[-0.152, 0.079]	2.764
FP -> RE	0.130	2.264	**0.024**	[0.017, 0.240]	2.764
MP -> IE	0.106	2.103	**0.035**	[0.009, 0.205]	2.764
FP -> IE	0.233	4.496	**<0.001**	[0.130, 0.333]	2.764
Indirect relationship					
MP -> RE ->PD	0.003	0.593	0.553	[-0.006, 0.014]	
FP -> RE ->PD	-0.010	1.705	0.088	[-0.022, -0.001]	
MP -> IE ->PD	0.032	2.047	**0.041**	[0.003, 0.063]	
FP -> IE ->PD	0.070	4.219	**<0.001**	[0.037, 0.103]	

Reported values include standardized path coefficients (β), t values, p values, variance inflation factors (VIF), and 95 percent bias corrected bootstrap confidence intervals based on 5,000 resamples. Variance inflation factor values below 3.00 indicate no multicollinearity concerns among predictor constructs. Indirect effects are considered statistically significant when the corresponding confidence intervals do not include zero.

Bold values indicate statistical significance (p < 0.05).

The model accounted for a substantial proportion of variance in psychological distress, with an R squared value of.373, indicating that parental psychological control and emotion regulation jointly explained 37.3 percent of the variance in psychological distress.

#### 3.3.1 Direct effect

With respect to direct effects, both paternal psychological control and maternal psychological control were positively and significantly associated with psychological distress. Paternal psychological control exerted a stronger effect on psychological distress [β = 0.319, t = 7.601, p < 0.001, 95% CI (0.238, 0.402)]. Maternal psychological control also showed a significant positive association with psychological distress [β = 0.162, t = 3.943, p < 0.001, 95% CI (0.079, 0.241)].

Emotion regulation strategies demonstrated differential associations with psychological distress. Expressive inhibition was positively related to psychological distress [β = 0.299, t = 10.826, p < 0.001, 95% CI (0.243, 0.352)]. In contrast, cognitive reappraisal was negatively associated with psychological distress [β = −0.074, t = 2.845, p = 0.004, 95% CI (−0.124, −0.023)].

Regarding predictors of emotion regulation strategies, paternal psychological control was positively associated with both cognitive reappraisal and expressive inhibition. The association with cognitive reappraisal was modest but statistically significant [β = 0.130, t = 2.264, p = 0.024, 95% CI (0.017, 0.240)]. Paternal psychological control was more strongly associated with expressive inhibition [β = 0.233, t = 4.496, p < 0.001, 95% CI (0.130, 0.333)]. Maternal psychological control was positively related to expressive inhibition [β = 0.106, t = 2.103, p = 0.035, 95% CI (0.009, 0.205)]. However, the association between maternal psychological control and cognitive reappraisal was not statistically significant [β = −0.038, t = 0.635, p = 0.526, 95% CI (−0.152, 0.079)].

#### 3.3.2 Mediation effects

Indirect effects were examined using a bootstrapping procedure with 5,000 resamples, and statistical significance was determined based on 95 percent confidence intervals. Expressive suppression was identified as a significant mediating pathway linking parental psychological control to psychological distress. Specifically, paternal psychological control indirectly increased psychological distress through expressive inhibition (β = 0.070, t = 4.219, p < 0.001, 95% CI [0.037, 0.103]). Maternal psychological control also demonstrated a significant indirect effect via expressive inhibition (β = 0.032, t = 2.047, p = 0.041, 95% CI [0.003, 0.063]).

In contrast, cognitive reappraisal did not significantly mediate the relationship between parental psychological control and psychological distress. The indirect effect of paternal psychological control through cognitive reappraisal was not significant (β = −0.010, t = 0.956, p > 0.05, 95% CI [−0.032, 0.012]). Similarly, the indirect effect of maternal psychological control through cognitive reappraisal was not significant (β = 0.003, t = 0.558, p > 0.05, 95% CI [−0.009, 0.017]).

## 4 Discussion

This study examined how parental psychological control influences psychological distress among university students exhibiting smartphone addiction, with a particular focus on the mediating roles of emotion regulation, namely cognitive reappraisal and expressive inhibition. The findings largely supported the proposed hypotheses and demonstrated that parental psychological control was significantly associated with both emotion regulation and psychological distress. In the present study, smartphone addiction was treated as a risk contextual background rather than a predictor within the analytical model, allowing for a focused examination of psychological mechanisms among individuals already vulnerable to emotional dysregulation.

### 4.1 Parental psychological control and psychological distress

Consistent with Hypothesis 1, both paternal and maternal psychological control were positively associated with psychological distress among smartphone addicted university students. Notably, paternal psychological control exerted a stronger direct effect on psychological distress than maternal psychological control. These results underscore the pivotal role of paternal control in shaping emotional responses among smartphone addicted students, aligning with prior studies (5, 42). This pattern highlights the differentiated roles of fathers and mothers in shaping emotional outcomes within the family system.

For university students with smartphone addiction, paternal psychological control may be particularly detrimental because these individuals often display heightened sensitivity to external control, impaired self-regulation, and increased reliance on smartphones as an emotional coping tool. Under such conditions, paternal authority and evaluative pressure may intensify feelings of autonomy frustration and psychological burden, thereby amplifying distress. This interpretation is consistent with ecological systems theory, which emphasizes that individual vulnerabilities interact with family level stressors to shape psychological outcomes.

### 4.2 Parental psychological control and emotion regulation

In line with Hypothesis 2, paternal psychological control was positively associated with both cognitive reappraisal and expressive inhibition, whereas maternal psychological control was positively related only to expressive inhibition. These differentiated associations suggest that fathers and mothers may activate distinct emotion regulation processes in their children.

In traditional Chinese culture, fathers are often perceived as primary authority figures (14, 43). Consequently, when confronted with paternal psychological control, students may attempt to cope by engaging in cognitive reappraisal to reinterpret paternal expectations and maintain internal balance. Cognitive reappraisal, as an adaptive strategy, enables individuals to modify emotional responses through cognitive restructuring, distinguishing it from the suppressive nature of inhibition (44, 45).

In contrast, maternal psychological control evokes different emotional responses. Given the typically stronger emotional bonds between mothers and children (5), maternal control is more likely to elicit feelings of guilt, obligation, or emotional dependency (46, 47). These relational pressures often prompt students to suppress emotional expression in order to preserve harmony. Among smartphone addicted students, expressive inhibition may be more easily activated because excessive smartphone use is frequently associated with avoidant coping, reduced emotional awareness, and a tendency to manage distress through disengagement rather than direct emotional processing.

### 4.3 Mediating role of emotion regulation

Consistent with Hypothesis 3, expressive inhibition significantly mediated the relationship between parental psychological control and psychological distress, whereas cognitive reappraisal did not exhibit a significant mediating effect. Expressive inhibition, as a maladaptive regulation strategy, is strongly linked to heightened psychological distress (48, 49). Unlike cognitive reappraisal, inhibition merely postpones emotional expression without resolving underlying emotional needs, leading to cumulative emotional strain over time.

This finding is consistent with evidence suggesting that inhibitory regulation can foster self-silencing processes, which in turn increase depressive symptoms, as demonstrated by Demir Kaya and Kaya (50). Similarly, Aksakal et al. (51) showed that inhibitory strategies are associated with higher levels of distress, whereas reappraisal is linked to more adaptive psychological outcomes. Within a smartphone addicted population, habitual reliance on digital devices for emotional escape may further reinforce inhibitory regulation patterns, thereby strengthening the indirect pathway from parental psychological control to psychological distress.

Taken together, these findings indicate that expressive inhibition represents a critical psychological mechanism through which parental psychological control translates into distress among smartphone addicted university students.

In conclusion, the findings revealed that inhibition plays a crucial mediating role in the relationship between parental psychological control and psychological distress. The emotional characteristics of parental control, particularly maternal, appear to more readily evoke inhibition, a coping strategy that, while effective in the short term, contributes to emotional accumulation and long-term distress. Moreover, excessive parental control may diminish students’ propensity to employ proactive strategies like cognitive reappraisal, thereby narrowing their emotion regulation repertoire.

## 5 Conclusion and recommendations

This study advances understanding of how family dynamics, emotion regulation processes, and digital contexts jointly influence psychological well-being. Specifically, the findings provide empirical support for the relevance of ecological systems theory in explaining how parental psychological control affects psychological distress through individual level regulatory processes.

Importantly, smartphone addiction in the present study functioned solely as an inclusion criterion rather than a modeled variable. It was conceptualized as a risk background condition, allowing the analysis to focus on psychological mechanisms within a vulnerable subgroup. Future research may extend this work by incorporating smartphone addiction as a moderating variable or by conducting multi group comparisons between addicted and non-addicted students to further clarify its role in shaping family related psychological processes.

From a practical perspective, the findings yield several implications. First, interventions targeting smartphone addiction should address not only excessive usage behaviors but also family interaction patterns, particularly paternal psychological control. Second, emotion regulation interventions that reduce reliance on inhibition and promote adaptive strategies may be especially beneficial for smartphone addicted students. Third, family education programs should encourage autonomy supportive parenting practices to mitigate the negative effects of psychological control. In psychological counseling settings, practitioners working with smartphone addicted university students may benefit from assessing parental control experiences and targeting expressive inhibition as a key therapeutic focus.

Several methodological constraints warrant consideration. To begin with, the use of a cross-sectional framework limits the ability to determine temporal or directional associations among parental psychological control, emotion regulation processes, and psychological distress. In addition, the exclusive dependence on self-reported data raises the possibility of measurement-related bias. Furthermore, the participant pool was confined to Chinese university students, which may restrict the extent to which these findings can be extrapolated to other developmental stages or sociocultural contexts.

In addition, it is important to note that smartphone addiction was treated as an inclusion criterion rather than an analytic variable in the current study. Conceptually, smartphone addiction can be understood as a high-risk condition that heightens vulnerability to psychological distress. From this perspective, the mechanisms identified in the present model may operate differently across varying levels of smartphone addiction severity. Future research could extend the current findings by incorporating smartphone addiction as a moderating variable or by conducting multi-group analyses to compare students with and without smartphone addiction. Such methodological approaches would facilitate a more refined assessment of whether the effects of parental psychological control are amplified among individuals at elevated risk and would yield clearer evidence regarding the role of family dynamics in problematic smartphone use among university students.

Finally, future studies are encouraged to incorporate additional psychological and contextual variables, such as self-esteem, self-control, social support, and sleep quality, and to employ longitudinal or multi-method designs to better capture developmental trajectories and causal processes. Expanding research across diverse cultural contexts would further clarify how sociocultural norms shape the psychological impact of parental psychological control in the digital age.

## Data Availability

The raw data supporting the conclusions of this article will be made available by the authors, without undue reservation.
